# Chinese consumers do not always respond to red: The influence of colors on perceived distance, spaciousness, and purchase intention of Chinese consumers

**DOI:** 10.3389/fpsyg.2022.1028425

**Published:** 2023-01-16

**Authors:** Dan Zhao, Fengwei Guan

**Affiliations:** ^1^College of Humanities, Jilin Agricultural University, Changchun, China; ^2^Changchun Institute of Optics, Fine Mechanics and Physics, Chinese Academy of Sciences (CAS), Changchun, China

**Keywords:** colors, perceived distance, perceived spaciousness, purchase intention, implicit association test, retail design

## Abstract

Many international firms hold a common stereotype about Chinese consumers’ color preference: culturally, red is their favorite color. However, many international firms (e.g., P&G, Ford, and Wal-Mart) do not use red as their theme colors when they run business in the Chinese market. To explain this interesting phenomenon, this study conducted three which include one IAT experiment and two scenari-based experiments to reveal less culture-laden influences of colors on people by examining the mediating effects of perceived spaciousness between colors and purchase intention. The results show that blue walls of a room make the room look more spacious than red ones and eventually increase consumers’ purchase intention. The perceived spaciousness is caused by the fact blue objects are perceived more distant than red ones. The findings indicate that culturally favorable color may not always be the most effective tool to increase consumers’ purchase intention. Hence, international firms should be extremely cautious when selecting a theme color in foreign markets.

## 1. Introduction

Many people hold a common stereotype about Chinese consumers’ color preference: culturally, red is their favorite color. This stereotype might be true since the Chinese even name a particular type of red (RGB: 230, 0, 0) Chinese red. Intuitively, firms should use red to please Chinese consumers and increase sales. However, many international firms (e.g., P&G, Ford, and Wal-Mart) do not customize their theme colors when they run business in the Chinese market. What is more interesting is that even Chinese firms do not adopt the color red as their theme color (e.g., Haier, China Construction Bank, and China Mobile) more often than any other colors although many Chinese believe that red can bring good luck, fortune, and happiness. To confirm this anecdotal observation, we conducted a pilot study of coding the theme colors of logos of the top 500 global brands in 2020. We found that eastern Asian brands do not differ from Western brands regarding theme color selection; the most common theme color is blue in both eastern Asian and western firms. The results of the pilot study indicate that Chinese enterprises do not tend to use red, a color commonly considered to entail positive cultural meanings, to improve brand value and consumer attitude. Instead, firms choose to use blue to strengthen their brand image. We argue that to explain these surprising results, it is necessary to reveal the possible positive influence of blue on Chinese consumers over red (Wang et al., 2014; Nutsford et al., 2016). In other words, this paper answers the question: Whether and why do Chinese consumers purchase more in a blue retail store than in a red one even though Chinese culturally favor red over blue? Without clearly understanding this question, international firms may have been using an inappropriate color in the Chinese market. Expanding to the global market, the findings of this study can offer valuable implications on whether a firm should use a color consistent with cultural meanings in a market to improve its image. To answer this question, we investigate the effects of blue and red on perceived spaciousness, which should not result from cultural learning but from instincts. Specifically, this paper examines the mediating effect of perceived spaciousness between colors (i.e., blue and red) and purchase intentions.

Both marketing scholars and practitioners emphasize the critical influence of colors on human emotions, evaluations of stores, and consumption behavior (Bitner, 1992; Hagtvedt and Brasel, 2017; van Esch et al., 2019; Cho and Suh, 2020; Ketron and Spears, 2020). Although many studies based on western culture have reached a consensus that people have more positive responses such as higher pleasure, trust, and purchase intention in a blue setting than in a red one (Bellizzi and Hite, 1992; Labrecque and Milne, 2012), there are still at least two research gaps to fill. First, it is underexplored why blue is commonly used even in cultures that favor other colors. Most studies about the influence of colors on people are based on associative learning theory, which proposes that meanings of colors are developed by people frequently associating colors with the common objects of the colors in a culture (Mehta and Zhu, 2009). Based on associative learning theory, people’s responses to a particular color are heavily culture-laden since common objects of the color may contain different meanings (Sakaki et al., 2014; Jonauskaite et al., 2019). In this sense, associative learning theory is limited in explaining the common use of blue in the Chinese market, which is commonly perceived as a culture that favors red. Therefore, it is necessary to explain the mechanism through which colors influence people’s responses from a less culture-laden perspective. Therefore, this study examines the influence of colors on perceived spaciousness (Payne, 1964), which in turn influences their purchase intention.

Second, previous research has primarily focused on the color of the products themselves instead of background colors (for exceptions for Bellizzi and Hite, 1992; Bagchi and Cheema, 2013). However, the background colors may unconsciously influence consumers’ decisions. Therefore, this study investigates how wall colors influence consumers’ perceived spaciousness of retail space and thus impact their purchase intention.

Perceived spaciousness reflects the extent to which people feel sufficiency in the space and freedom to move in an environment (Eroglu et al., 2005), and it impacts various human judgments and behaviors (Dennis et al., 2002; Kesari and Atulkar, 2016; Presti et al., 2022). By investigating the influence of colors on perceived spaciousness and purchase intention, the present study makes twofold contributions to existing literature. First, our findings enrich our understanding of the influence of colors on people’s responses. Previous studies have predominantly focused on human behavior through psychological mechanisms such as emotions, and mood, among others (Su et al., 2019; van Esch et al., 2019). However, these psychological responses caused by colors should vary across cultures because colors entail different meanings in different cultures (Madden et al., 2000). Different from previous studies, the present study investigates a less culture-laden outcome, which is perceived spaciousness. By doing so, our findings help explain why some colors have constant influences across cultures by supplying the missing piece of the puzzle of outcomes of colors. Thus, our results help to explain why some colors (e.g., blue) are predominantly used across countries, firm types, product categories, and so on.

Second, the results offer insight into retail space design. Retail space determines the scale of inventory and shelf space in a retail store and consumers’ shopping experience. As rentals grow more expensive each day and because of inflations, how to increase consumers’ perceived spaciousness in a given space is a vital question. In addition, when consumers perceive that a retail store is spacious, they should feel less crowded and have a better shopping experience (Ahmed and Ting, 2020). Our findings show that blue walls make consumers feel a store is more spacious than red walls. Changing the color of walls is an easy and inexpensive undertaking, but it decreases consumers’ perceived crowdedness so that retailers can put more shelves in a given space. Therefore, retailers could increase the effectiveness of their space by changing the color of their walls.

In the remainder of the paper, we first briefly review relevant literature and propose hypotheses. Then, we use an implicit experimental design to examine a less culture-laden influence of colors on people’s perceptions. Next, we use two explicit experimental designs to examine the influences of colors on perceived spaciousness, which in turn increases consumers’ purchase intention. Finally, we conclude the study by discussing implications, limitations, and future research directions.

## 2. Theoretical background and hypotheses

### 2.1. The influence of red and blue and associative learning theory

Colors have attracted plenty of research attention in the extant literature and have been demonstrated to influence human behaviors significantly (e.g., Bottomley and Doyle, 2006; Lick et al., 2017; Kunz et al., 2020; Wang et al., 2021). For example, Hill and Barton (2005) have shown that athletes wearing red uniforms are more likely to win sports competitions because red can evoke people’s aggressiveness more than blue. In addition, bright colors (e.g., white, pink) mainly elicit positive emotions, while dark colors (e.g., brown, black) mainly evoke negative emotional associations (Hemphill, 1996). Since blue and red lie on the two ends of the color spectrum based on wavelength, many studies have focused on the contrasting effects of blue versus red on human psychological and behavioral responses (e.g., Chylinski et al., 2015; van Esch et al., 2019). Previous studies have found that blue is more related to peace, trust, pleasantness, attractiveness, satisfaction, and competence, while red is more related to excitement, power, depression, dissatisfaction, and repulsion (Stone and English, 1998; Stone, 2003; Hill and Barton, 2005; Baxter et al., 2018; van Esch et al., 2019). For example, because red can evoke human aggressiveness and desires to obtain objects, people tend to offer a lower price in negotiations but a higher price in bidding to strike a deal (Hill and Barton, 2005; Bagchi and Cheema, 2013). In a retail store, consumers tend to purchase more merchandise and are less likely to postpone their purchase decisions when the background color of the store is blue rather than red (Hsieh et al., 2018). Additionally, people have higher brand trust and pleasure in a blue retail setting than in a red one (Bellizzi and Hite, 1992; Su et al., 2019). Generally, human’s tendency to approach (versus avoid) could be activated by blue (versus red); thus, people purchase more products in a blue retail store than in a red one (Bellizzi and Hite, 1992; Mehta and Zhu, 2009).

Associative learning theory has been commonly used to explain the different influences of blue and red on people’s responses. Associative learning theory proposes that meanings of colors are developed by people frequently associating colors with the common objects of the colors in a culture (Mehta and Zhu, 2009). In human’s daily lives, common blue objects are sky and ocean, which are usually viewed as approachable in many cultures Therefore, people tend to assign positive meanings to blue (Su et al., 2019). By contrast, common red objects are fire and blood, which are usually viewed as unapproachable in many cultures (Su et al., 2019). Therefore, people tend to associate red with negative meanings (Bellizzi et al., 1983). The associations between blue (versus red) and positive (versus negative) meanings are particularly salient in English-speaking countries (Elliot and Maier, 2007). In English, “seeing red” can be used to represent anger (Fetterman et al., 2012). And in those countries, teachers usually use red ink to mark mistakes, and red warning signs indicate stop, but common blue objects are not associated with negative meanings (Elliot and Maier, 2007; Mehta and Zhu, 2009). The associations also reflect in human behavioral responses and information processing. For example, Kuhbandner and Pekrun (2013) have shown that when negative words are shown in red rather than blue, it is easier for people to memorize those words. By contrast, when positive words are shown in green than red, people can recall words more easily (Kuhbandner and Pekrun, 2013). All these studies have consistently shown that people have different emotional, psychological, and behavioral responses in an environment with different colors since people associate the colors with different meanings (Shanks, 2010).

### 2.2. The influence of red and blue on Chinese consumers

It is noticeable that the meanings that the aforementioned common red or blue objects represent might differ across cultures. Specifically, fire and blood usually symbolize dangers in Western culture. By contrast, in traditional Chinese culture, fire does not represent danger but something that could scare away an imaginary monster, “Nian, “which is the figure referenced in the Chinese Spring Festival (Wen et al., 2014). This cultural figure is an example that can be used to explain why the Chinese use red to pay for good luck, fortune, peace, and safety. In light of this cultural perspective, blue may not evoke more positive responses than red for Chinese, and instead, red should be more effective in stimulating Chinese consumers’ positive responses. Contrary to this cultural intuition, our pilot study shows that large and reputable Chinese firms use blue more than red in their brand esthetics, a phenomenon that existing studies have not thoroughly investigated. To fill this gap, it is necessary to discover the possible positive influence of blue over red on Chinese consumers’ responses.

### 2.3. The influence of red and blue on perceived spaciousness

The present study takes a lens of perceived spaciousness to explain why Chinese might purchase more in a blue rather than a red store even though Chinese culture favors the color red. This study focuses on perceived spaciousness because it is a vital concept in retailing studies and substantially influences people’s shopping experiences (Aydinli et al., 2020). Specifically, perceived spaciousness can alleviate people’s negative experiences caused by crowding, improve people’s sense of control over the environment (Hui and Bateson, 1991), reduce anxiety and stress (Maeng et al., 2013), increase social interaction motivation (Sommer, 2009), and mitigate avoidance motivation (Eroglu and Machleit, 1990). More importantly, the sense of spaciousness is unlikely to be influenced by culture since it results from eyes processing space. Therefore, this less culture-laden variable may help explain why Chinese firms do not commonly use red, a theme color that is well aligned with their culture.

A series of studies have shown that objects’ surface colors affect people’s judgments of size and distance (Payne, 1964). Objects of the same size may appear to have different sizes due to the reflections of colors. For example, to make the three blocks of different colors (blue, white, and red) on the French national flag look isometric, the actual proportion of the Blue, White, and Red color blocks is adjusted to 30:33:37 (Harada, 2009) since blue objects look bigger than their actual sizes. Indeed, previous studies indicate that objects whose surface color is red look closer than those of blue. Therefore, studies refer to red and warm colors as advancing colors, which refer to colors that make objects closer than they actually are, and blue and cool colors as retiring colors, which refer to colors that make objects look farther than they actually are (Luckiesh, 1918; Goethe, 1982). At least two theories can explain this phenomenon.

Firstly, sensitive cones in human retinas distinctly respond to light with different wavelengths. Specifically, sensitive cones are more susceptible to long-wavelength light (e.g., red) than short-wavelength (e.g., blue). The difference in the sensitivity to light and colors is so significant as to bias human perception of distance (Livingstone, 2002). The less sensitive cones are to colors, the more distant the colors appear. Therefore, objects with cold colors (e.g., blue) seem more distant than the ones with warm colors (e.g., red).

Secondly, the human eye’s operational mechanism can be used to explain why blue objects look more distant than red ones (Sundet, 1978). Previous studies show that short-wavelength (e.g., blue) light refracts in the eye’s optical medial more than a long one (e.g., red) does, so a short-wavelength color looks more distant than a long-wavelength does (Olguntürk, 2016). Specifically, a short wavelength’s natural focal point is in front of the retina, while the long wavelengths are slightly behind the retina, so the eye’s lens must adjust to produce sufficient refractive power to focus on long-wavelength light (Sundet, 1978). Therefore, blue objects look more distant than red ones. In sum, we hypothesize that:

*H1*: Blue objects look more distant than red objects.

In a retail space, the walls of a retail store define its boundaries, and the distance of walls to observers should impact their evaluations of a room’s spaciousness. Consistent with H1 that hypothesizes blue objects look more distant than red objects, we propose that a room with blue walls should look more spacious than a room with red walls. If walls look distant to consumers, they will make a room look more spacious, and vice versa. In addition, studies in the fields of architecture and environmental design also suggest that blue walls make a room look more spacious than red ones. Specifically, some studies indicate that walls of warm colors (e.g., red) can increase consumers’ arousal, so consumers would perceive a room as smaller than it actually is. By contrast, walls of cool colors (e.g., blue) make consumers calm, so consumers perceive a room as spacious (Bellizzi et al., 1983; Crowley, 1993; Stone, 2003; Yildirim et al., 2007, 2012; Odabaşioğlu and Olguntürk, 2015).

We particularly focus on the colors of walls rather than other objects in a room such as ceiling and doors for two reasons. First, the height of walls is usually at human’s eye height. It is noticeable that perceived egocentric distance, which is defined as the distance from objects to observers, is influenced by eye height (Ooi et al., 2001). Specifically, people feel objects are more distant from a lower eye height than a regular or higher eye height. Therefore, eye height also influences the perceived spaciousness of a room (Leyrer et al., 2011). To control the confounding effects of eye height, we focus on how the colors of walls in a retail store influence people’s perceived spaciousness of the store since walls in retail space are usually at human’s eye height.

Second, previous studies suggest that the colors of walls influence people’s perceived spaciousness of a room. For example, by changing the lighting color of the walls, Odabaşioğlu and Olguntürk (2015) show that people’s perceived spaciousness of a room is highest when the lighting color is white, followed by green and red. Since human eyes can discern the differences between lighting and wall colors (Burns and Caughey, 1992; Odabaşioğlu and Olguntürk, 2015;), it is necessary to examine how blue and red walls of a retail space influence people’s perceived spaciousness and shopping experience.

In sum, we argue that since blue walls are perceived as more distinct than red walls, a room with blue walls should be perceived as more spacious than a room with red walls. Formally, we hypothesize that:

*H2*: People perceive a retail store with blue walls to be more spacious than a retail store with red walls.

As a result of evolution, humans, like other species, prefer spacious space because humans usually feel threatened, urgent, and uncomfortable when space decreases (Hediger, 1955; Hill and Barton, 2005). Therefore, in a retail setting, perceived spaciousness could mitigate consumers’ perceived threats, tension, anger, disgust, and contempt and increase consumers’ joy and interest (Eroglu et al., 2005; Stamps, 2011). For example, overcrowding reduces consumers’ satisfaction with their shopping experiences by hindering them from quickly finding the products they aim to buy (van Rompay et al., 2012). When consumers are in a crowded environment, their avoidance motivation can be evoked; accordingly, their willingness to stay may decrease (Harrell et al., 1980; Eroglu and Machleit, 1990). Under this circumstance, consumers’ purchase of products, especially impulsive products, will reduce (Milgram, 1970; Harrell et al., 1980). Indeed, Hui and Bateson (1991) have found that the high density of a retail store decreases consumers’ perceptions of control, which in turn decreases pleasure and purchase intention. By contrast, in a spacious shopping environment, consumers usually feel more relaxed and pleasant, tend to perceive more value, and have higher satisfaction and purchase intention (Yüksel, 2009; Pons et al., 2016).

In sum, retail stores should increase the effectiveness of a given space by manipulating consumers’ perceived spaciousness, which in turn can evoke more purchases (Machleit et al., 2000). As consumers feel that a blue retail store is more spacious than a red one, consumers should have higher purchase intention in a blue versus red retail store. According to this logic, we hypothesize:

*H3*: Perceived spaciousness mediates the relationship between blue (versus red) and purchase intention.

## 3. Study 1

### 3.1. Experimental stimuli and process

The objective of study 1 is to test *H1*, which states that blue objects look more distant than red ones to people. To test the hypothesis, we conducted an implicit association test (IAT). IAT has been widely used in the field of marketing to reveal an implicit relationship between two variables with influences of contextual factors removed (e.g., Bar-Anan et al., 2006; Lee et al., 2014; Su et al., 2019). Since this study aims to reveal a less culture-laden influence of colors on human perceptions, an IAT is appropriate. In an IAT task, a participant is asked to group stimulus words with target words based on the proximity of meanings of the words. The time to complete the task is used to gauge the strength of the relationship between two variables. Specifically, the shorter the time, the stronger the relationship between the two variables.

In this study, a participant is exposed to one stimulus word at a time, synonymous with proximity or distance, and two target words (i.e., proximity and distance). Then, they are asked to group the stimulus word with one of the two target words. Based on the results of a pretest, we used eight stimulus words synonymous with proximity (i.e., “一箭之地,” “一墙之隔,” “一步之遥,” “摩肩接踵,” “比邻而居,” “鸡犬相闻,” “比肩而立,” “近在咫尺”) and eight stimulus words synonymous with distance (i.e., “远隔重洋,” “千山万水,” “山高水远,” “天各一方,” “天南地北,” “日东月西,” “相去万里,” “天涯海角”). In addition, we included six distractor words, three of which were related to red (i.e., “万紫千红,” “粉墙朱户,” “红装素裹”) and three of which were related to blue (i.e., “碧空如洗,” “山青水碧,” “青出于蓝”; See Appendix 1 for the translations).

**Figure 1:**
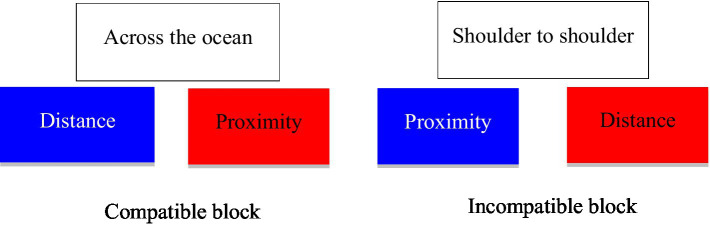
Experimental stimuli used in the Implicit Association Test (shown in Chinese in study 1).

We recruited 323 participants for the IAT from a Chinese public university. The experiment was conducted *via* four blocks. The first two blocks were practice blocks. Specifically, in the first block, the participants were asked to categorize each distractor word into a “blue” or a “red” group, and in the second block to categorize each stimulus word into a “proximity” or “distance” group. The third block was a compatible block in which “distance” was paired with “blue” by putting “distance” in a blue box while “proximity” was paired with “red” by putting “proximity” in a red box. The fourth block was an incompatible block in which “proximity” was paired with “blue” and “distance” was paired with “red” as target words (see Figure 1).

We randomly chose eight proximity/distance-related words (i.e., “天南地北,” “一步之遥,” “日东月西,” “远隔重洋,” “一墙之隔,” “相去万里,” “比邻而居,” “鸡犬相闻”) and three color-related words (i.e., “青出于蓝,” “万紫千红,” “粉墙朱户”) as stimulus words for the compatible group. The remaining eight proximity/distance-related words (i.e., “天各一方,” “千山万水,” “天涯海角,” “近在咫尺,” “比肩而立,” “山高水远,” “一箭之地,” “摩肩接踵”) and three color-related words (i.e., “红装素裹,” “碧空如洗,” “山青水碧”) were used as stimulus words in the incompatible block. In the third and fourth blocks, participants were asked to categorize each stimulus word into one of the target words as shown in Figure 1. To avoid order effects, we randomized the occurrence of all stimulus words and the order of options of target words in the compatible and incompatible blocks.

### 3.2. Data analysis and results

The data was analyzed through three stages. Firstly, consistent with Su et al. (2019), we deleted the cases if more than fifty seconds were spent on categorizing any one word because it is not reasonable to spend more than that on a word categorization task. At this stage, 268 responses remained for further analysis. Secondly, as Greenwald et al. (2003) suggested, we implemented a 600-millisecond penalty for incorrect answers. Lastly, we conducted a paired t-test on the mean time of categorizing words in the compatible versus incompatible block. The results showed that response time was significantly shorter in the compatible block pairing “blue” and “distance” (*M* = 2.340 milliseconds, *SD* = 1.191 milliseconds) than in the incompatible block pairing “blue” and “proximity” (*M* = 2.516 milliseconds, *SD* = 1.271 milliseconds; *t* (267) = 2.277, *p* < 0.05). The results showed that the participants were able to categorize stimulus words significantly faster when blue rather than red was accompanied by “distance,” suggesting that blue is related more strongly to distance than is red, supporting H1.

The results of study 1 indicate that blue is more related to distance than is red. Therefore, blue objects should look more distant than should red ones. To further provide empirical evidence from a real-world perspective, study 2 conducted an explicit experiment to test H2.

## 4. Study 2

The objective of study 2 is to provide more empirical evidence for the relationship between colors and perceived distance *via* an explicit experiment. If blue objects look more distant than red ones, blue walls in a retail store should make people perceive more spaciousness than should red walls, as H2 hypothesizes.

### 4.1. Experimental stimuli and process

**Figure 2:**
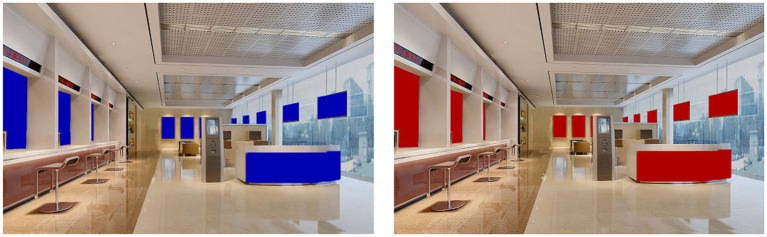
Experimental stimuli in study 2.

In study 2, we recruited 236 participants from an internet-based data collection platform based in China. We paid each participant 7 yuan (about $1) as compensation. After deleting cases that failed in two attention-check questions, 188 cases remained for data analysis. One of the attention-check questions asked what the theme color of the bank was, so this question also checked manipulation. The participants were asked to imagine that they were going to a bank to open a checking account. We then randomly exposed participants to a picture of a bank lobby with either blue or red walls. Specifically, 95 participants saw a bank lobby with blue walls, and 93 participants saw a bank lobby with red walls (see Figure 2).

After the participants had seen the picture, we measured their perceived spaciousness of the bank lobby. Consistent with Yüksel (2009), we asked the participants to rate the spaciousness of the room by answering a seven-point bipolar question from confined (1) to spacious (7). Finally, we collected the participants’ demographic information. Consistent with existing cross-cultural studies (e.g., Wang et al., 2021), two bilingual researchers used a standard back-translation approach to guarantee the languages were appropriately translated from English to Chinese.

### 4.2. Data analysis and results

We conducted an independent t-test to compare people’s perceived spaciousness of the bank lobby with blue versus red walls. The results show that the room with blue walls (*M* = 6.063, S.E. = 0.810) is perceived as more spacious than the room with red walls (*M* = 5.796, S.E. = 1.239), and the difference is statistically marginally significant (*t* (158) = 1.749, *p* < 0.10). The results show that blue walls make a room look more spacious than red walls because blue walls look more distant to participants than red walls, supporting H2.

## 5. Study 3

The main objective of study 3 is to test H3, which hypothesizes the mediating effects of perceived spaciousness between colors and purchase intention. Specifically, study 3 extends study 2 in three ways. First, we test the relationships between colors and perceived spaciousness in a different retail context (i.e., a restaurant) from study 2 (i.e., a bank) to increase the generalizability of the findings. Second, study 3 tests the mediating effects of people’s perceived spaciousness of a retail store between colors and purchase intentions and provides pertinent guidance on retail store design, as H3 hypothesizes. Third, to increase the robustness of the less culture-laden influence of colors on purchase intention, study 3 adds an important culture-laden variable, approach motivation, as a control variable into the regression model. Based on associative learning theory, blue evokes approach motivation, while red evokes prevention motivation (Mehta and Zhu, 2009; Su et al., 2019), which might vary across cultures. To validate the culture-free influence of colors, the approachability of a store was added as a control variable.

### 5.1. Experimental stimuli and process

**Figure 3:**
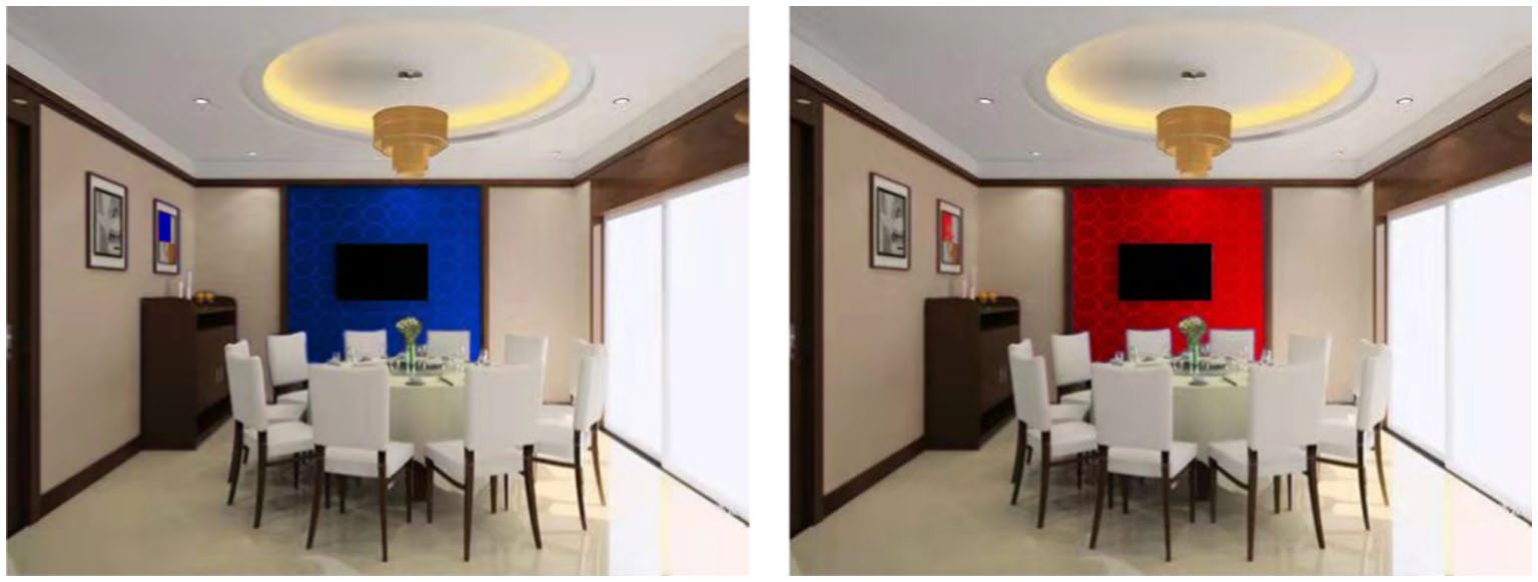
Experimental stimuli in study 3.

In study 3, we recruited 312 participants from the same data collection platform as in study 2. We paid each participant 7 yuan (about $1) as compensation. After deleting those that failed in two attention-check questions, 299 cases remained for data analysis. Consistent with experiment 2, one of the attention-check questions also checked manipulation by asking what the theme color of the restaurant is. In this experiment, participants were asked to imagine that they were heading for dinner with a friend; they were then randomly exposed to a picture of a restaurant with either red or blue walls. Specifically, 150 participants saw a restaurant with blue walls, and 149 participants saw a restaurant with red walls (See Figure 3).

After the participants had seen the picture, we asked them to rate the spaciousness of the room as we did in study 2. Then we measured the approachability of the restaurant using eight items (Cronbach’s α = 0.700): “Would you enjoy dining in this restaurant?” “How much time would you like to spend in this restaurant?” “Would you avoid ever having to return to this store? (R)” “Is this a place in which you would feel friendly and talkative with a stranger who happens to be near you?” “Would you want to avoid looking around or exploring this environment? (R)” “Do you like the environment of this restaurant?” “Is this a place where you might try to avoid other people and avoid having to talk to them? (R)” and “Is this the sort of place where you might end up spending more money than you originally set out to spend?” (Donovan and Rossiter, 1982). Next, we used a well-established seven-point scale to measure patronage intentions (Martins et al., 2019). They are: “I find purchasing product/service at this restaurant to be worthwhile,” “I will frequently purchase product/service advertised at this restaurant in the future,” and “I will strongly recommend others to purchase product/service advertised at this restaurant” (Cronbach’s *α* = 0.725). Finally, we collected the participants’ demographic information. To guarantee that the words were accurately translated into Chinese, two bilingual researchers followed a back-translation approach to translate scales into Chinese.

The measurement has acceptable convergent validity. Composite reliability for the model constructs ranged between 0.727 and 0.728, and Cronbach’s *α* ranged between 0.700 and 0.725, indicating good reliability. Moreover, the average variance extracted (AVE) was greater than 47.20% for all constructs, and all correlation coefficients between the two constructs were less than the square root of AVEs of each construct. The results provided support for discriminant validity. For the results of validity, reliability, and correlations between the constructs, see Appendix 2.

### 5.2. Data analysis and results

#### 5.2.1. Perceived spaciousness and purchase intentions

We conducted a series of independent t-tests on perceived spaciousness, purchase intentions, and approachability of the restaurant. Moreover, the participants feel that the restaurant is more approachable when the walls are blue (*M*_blue_ = 5.397, S.E._blue_ = 0.700) than when walls are red [*M*_red_ = 5.188, S.E._red_ = 0.827; *t* (290) = 2.265, *p* < 0.01]. The results show that the participants feel that the restaurant is more spacious when walls are blue (*M*_blue_ = 5.946, S.E._blue_ = 1.025) than when walls are red [*M*_red_ = 5.573, S.E._red_ = 0.787;*t* (279) = 3.530, *p* < 0.01]. Finally, the participants’ purchase intentions are different when walls are blue (*M*_blue_ = 5.763, S.E._blue_ = 0.906) than when they are red (*M*_red_ = 5.582, S.E._red_ = 0.806), and the difference is statistically marginally significant [*t* (293) = 1.822, *p* < 0.10].

#### 5.2.2. Mediation

To test the mediating effect of perceived spaciousness between colors and purchase intention, we conducted a bootstrap estimate with 10,000 re-samples through model 4 in SPSS PROCESS (Hayes, 2013), with color as the independent variable (1 = red, 2 = blue), purchase intention as the dependent variable, spaciousness as the mediator, and approachability as a control variable. The results show that the indirect effect is significant (*B* = 0.034, S. E. = 0.020, 95% confidence interval = 0.001, 0.147), indicating the mediation. Specifically, the results show that when the walls of a restaurant are blue rather than red, people feel the restaurant is more spacious, which in turn increases people’s purchase intentions when controlling the approachability of the restaurant.

## 6. General discussion

### 6.1. Theoretical and managerial implications

Existing studies consistently show that people respond more positively in a blue retail store than in a red one. For example, some studies show that consumers in blue environments will purchase more products than in red ones (Bellizzi and Hite, 1992). The rationale behind this relationship is that people usually associate blue with relaxing objects but red with depressing objects. As a result, red tends to evoke consumers’ avoidance motivation, but blue approach motivation (Mehta and Zhu, 2009; Su et al., 2019). However, these findings cannot explain why blue has been commonly used by firms in the Chinese market that predominantly favors red over blue. To solve this puzzle, the present study focuses on a less culture-laden mediator: perceived spaciousness. Specifically, through three studies, this paper demonstrates that blue objects look more distant compared to red ones, so blue walls of a retail store make a store appear more spacious than red walls, and perceived spaciousness increases people’s purchase intentions.

The findings make significant contributions to the current literature in at least two ways. First, the findings enrich the literature on color research. Color is a vital marketing tool to improve people’s evaluations of a store (Babin et al., 2003; Ettis, 2017; Roschk et al., 2017). Admittedly, a color may have different meanings in different cultures because of social learning (Madden et al., 2000). Our findings show that colors also produce culture-free influences such as perceived spaciousness besides culture-laden influences. The findings explain why Chinese firms prefer using blue over red, even though their culture favors red. However, in no way do our results suggest that all firms in the Chinese market should use blue in aesthetic designs. Color selection entails a complicated process, which involves a meshing with firms’ theme color, avoiding a clash with other colors, and so on. Our results only provide an alternative perspective for firms regarding their color choices.

Second, the findings enrich our understanding of perceived crowdedness, which has attracted plenty of research attention in the field of retailing (Baker and Wakefield, 2012; Rayburn and Voss, 2013). Since perceived crowding reduces consumers’ satisfaction with shopping experience, perceived value, and purchase intention, many scholars have devoted effort to looking for approaches to reducing consumers’ perception of crowding (Machleit et al., 2000; Mehta et al., 2013). Our results provide an easy and inexpensive undertaking to reduce consumers’ perception of crowding, which is to change the color of walls in retail spaces. By painting blue walls, retailers can engender a feeling of spaciousness, thus increasing consumers’ purchase intention.

From a managerial perspective, our results provide insight into retail design. To improve consumers’ shopping experience, retailers meticulously choose theme colors. For example, U.S.-based Walmart uses blue while U.S.-based Target uses red as theme color. Our results demonstrate some benefits of using blue rather than red, which are blue walls make a retail space appear more spacious and so increase consumers’ purchase intentions. Therefore, retailers may want to paint their walls blue if they want consumers to perceive higher spaciousness. However, our results do not suggest that red is a bad choice for consumers since red could evoke consumers’ excitement (Labrecque and Milne, 2012). Retailers should choose a color based on their purposes, missions, and values. For example, in many cases, entertainment firms may want to use red since they want consumers to feel excited and aroused.

### 6.2. Research limitations and future research directions

There may exist a few limitations in this study, reducing the generalizability of the findings. Firstly, this research does not consider demographic characteristics such as age and gender. Researchers mentioned that consumers of different genders and ages have different responses to crowdedness (Baker and Wakefield, 2012). Future research could test whether the proposed relationships hold constant across genders and ages.

Secondly, this research overlooks possible positive influences of feeling crowded. Previous studies demonstrate that consumers with hedonic motivation are likely to have more positive responses to crowding rather than a spacious environment (Yildirim et al., 2007). Therefore, feeling confined is not always a bad thing. Under some circumstances, people might feel cozy in a less spacious space, and in this scenario, using the red color might be a better choice. Therefore, future research should investigate under what circumstances red makes people feel cozy rather than cramped.

Thirdly, this study tested the influence of colors on perceived spaciousness and purchase intention in hypothetical scenarios. Therefore, external validities of the results may be limited. Therefore, future research should investigate how blue versus red decorations in a retail store influence people’s responses.

Fourthly, studies in the field of architectural design suggest that the colors of walls, ceilings, and other decorations should produce different influences on people (Hogg et al., 1979). Our findings show that blue walls appear farther away than red ones. Therefore, when a red table is placed in the center of a room, people should feel the table is close to them. As a result, they may perceive the rest of the surroundings as spacious. Therefore, future research must examine whether red furniture makes a room look more spacious than blue furniture. In addition, people’s perceptions of colors also depend on the relationship between objects and eye height. Therefore, wall color should produce influences on people different from those produced by floor or ceiling colors, which is an interesting question that future research must investigate.

Finally, it is interesting to investigate under what conditions Chinese feel dangerous or safe in a red context. Since fire represents both safety and danger for Chinese people, it is important to investigate what marketing tactics could trigger their perceptions of danger or safety. Using red inappropriately in the Chinese market might lead to disastrous outcomes if people’s perception of danger is evoked.

## Data availability statement

The original contributions presented in the study are included in the article/supplementary material, further inquiries can be directed to the corresponding author.

## Ethics statement

The studies involving human participants were reviewed and approved by College of Humanities, Jilin Agricultural University. The patients/participants provided their written informed consent to participate in this study.

## Author contributions

All authors listed have made a substantial, direct, and intellectual contribution to the work and approved it for publication.

## Funding

This work was supported by Vocational Education and Adult Education Teaching Reform Research Project of Jilin Province (2022ZCY246), The 14th Five Year Plan for Educational Science in Jilin Province (GH22603), and The 14th Five Year Plan for Educational Science in Jilin Province (ZD22043). The funder is Jilin Provincial Department of Education.

## Conflict of interest

The authors declare that the research was conducted in the absence of any commercial or financial relationships that could be construed as a potential conflict of interest.

## Publisher’s note

All claims expressed in this article are solely those of the authors and do not necessarily represent those of their affiliated organizations, or those of the publisher, the editors and the reviewers. Any product that may be evaluated in this article, or claim that may be made by its manufacturer, is not guaranteed or endorsed by the publisher.
