# Supplementary material for: Chinese consumers do not always respond to red: The influence of colors on perceived distance, spaciousness, and purchase intention of Chinese consumers
Source: Front Psychol. 2023 Jan 16;13:1028425. doi: 10.3389/fpsyg.2022.1028425 (PMC9887659; doi:10.3389/fpsyg.2022.1028425)
Supplement: Supplementary file 1 [file Table_1.docx]

**Appendix 1.** Translations of Words in Experiment 1

| **Categories** | **Chinese** | **English** |
| --- | --- | --- |
| **Synonyms of proximity** | 一箭之地 | distance of only one arrow shot |
|  | 一墙之隔 | separated by only one wall |
|  | 一步之遥 | distance of only one step |
|  | 摩肩接踵 | shoulder to shoulder |
|  | 比邻而居 | live next to the neighbor |
|  | 鸡犬相闻 | close enough to hear neighbors' chickens and dogs |
|  | 比肩而立 | stand next to the other person's shoulders |
|  | 近在咫尺 | so close at a distance of several inches |
| **Synonyms of distance** | 远隔重洋 | across the ocean |
|  | 千山万水 | separated by thousands of mountains and rivers |
|  | 山高水远 | high mountains and far rivers |
|  | 天各一方 | on two separate sides of the sky |
|  | 天南地北 | the south of the sky and the north of the earth |
|  | 日东月西 | the sun is on the east and the moon is on the west |
|  | 相去万里 | a distance of ten thousand miles |
|  | 天涯海角 | the end of the sky and the corner of the sea |
| **Synonyms of red** | 万紫千红 | very purple and red |
|  | 粉墙朱户 | pink walls and red houses |
|  | 红装素裹 | red sun and white snow |
| **Synonyms of blue** | 碧空如洗 | the sky is so blue as if it has been washed |
|  | 山青水碧 | green mountains and blue rivers |
|  | 青出于蓝 | indigo-blue stems from blue |

**Appendix 2.** Measurement and descriptive statistics

|  | **Perceived spaciousness** | **Approachability** | **Patronage intention** |
| --- | --- | --- | --- |
| **Perceived spaciousness** | N.A. |  |  |
| **Approachability** | 0.455** | *0.716* |  |
| **Patronage intention** | 0.400** | 0.656** | *0.687* |
|  |  |  |  |
| **Mean (S.D.)** | Study 2: 5.964 (0.988)  Study 3: 5.774 (0.978) | Study 3: 5.663 (0.868) | Study 3: 5.340 (0.750) |
|  |  |  |  |
| **Cronbach**'**s α** | N.A. | 0.700 | 0.725 |
| **Composite reliability** | N.A. | 0.728 | 0.727 |
| **Average variance extracted (AVE)** | N.A. | 0.512 | 0.472 |

Note: The value on the diagonal are square roots of AVE. **: p < 0.01
